# The novel S59P mutation in the *TNFRSF1A* gene identified in an adult onset TNF receptor associated periodic syndrome (TRAPS) constitutively activates NF-κB pathway

**DOI:** 10.1186/s13075-015-0604-7

**Published:** 2015-04-03

**Authors:** Eliana Greco, Ada Aita, Paola Galozzi, Alessandra Gava, Paolo Sfriso, Ola H Negm, Patrick Tighe, Francesco Caso, Filippo Navaglia, Emanuela Dazzo, Marzia De Bortoli, Alessandra Rampazzo, Laura Obici, Simona Donadei, Giampaolo Merlini, Mario Plebani, Ian Todd, Daniela Basso, Leonardo Punzi

**Affiliations:** University of Padova, Rheumatology Unit, Department of Medicine - DIMED, Via Giustiniani 2, 35128 Padova, Italy; University of Padova, Laboratory Medicine, Department of Medicine - DIMED, Via Giustiniani 2, 35128 Padova, Italy; School of Life Sciences, The University of Nottingham, Queen’s Medical Centre, Derby road, NG7 2UH Nottingham, UK; Medical Microbiology and Immunology Department, Faculty of Medicine, Mansoura University, Elgomhouria Street, 35516 Mansoura City, Egypt; Institute of Neuroscience of the National Research Council, Section of Padova, Corso Stati Uniti, 4, 3512 Padova, Italy; Department of Biology, University of Padova, Via U. Bassi, 58/B, 35121 Padova, Italy; Amyloidosis Research and Treatment Center, Biotechnology Research laboratories, Fondazione IRCSS Policlinico San Matteo and University of Pavia, Viale Camillo Golgi 19, 27100 Pavia, Italy

## Abstract

**Introduction:**

Mutations in the *TNFRSF1A* gene, encoding tumor necrosis factor receptor 1 (TNF-R1), are associated with the autosomal dominant autoinflammatory disorder, called TNF receptor associated periodic syndrome (TRAPS). TRAPS is clinically characterized by recurrent episodes of long-lasting fever and systemic inflammation. A novel mutation (c.262 T > C; S59P) in the *TNFRSF1A* gene at residue 88 of the mature protein was recently identified in our laboratory in an adult TRAPS patient. The aim of this study was to functionally characterize this novel *TNFRSF1A* mutation evaluating its effects on the TNF-R1-associated signaling pathways, firstly NF-κB, under particular conditions and comparing the results with suitable control mutations.

**Methods:**

HEK-293 cell line was transfected with pCMV6-AC construct expressing wild-type (WT) or c.262 T > C (S59P), c.362G > A (R92Q), c.236C > T (T50M) *TNFRSF1A* mutants. Peripheral blood mononuclear cells (PBMCs) were instead isolated from two TRAPS patients carrying S59P and R92Q mutations and from five healthy subjects. Both transfected HEK-293 and PBMCs were stimulated with tumor necrosis factor (TNF) or interleukin 1β (IL-1β) to evaluate the expression of TNF-R1, the activation of TNF-R1-associated downstream pathways and the pro-inflammatory cytokines by means of immunofluorescent assay, array-based technique, immunoblotting and immunometric assay, respectively.

**Results:**

TNF induced cytoplasmic accumulation of TNF-R1 in all mutant cells. Furthermore, all mutants presented a particular set of active TNF-R1 downstream pathways. S59P constitutively activated IL-1β, MAPK and SRC/JAK/STAT3 pathways and inhibited apoptosis. Also, NF-κB pathway involvement was demonstrated *in vitro* by the enhancement of p-IκB-α and p65 nuclear subunit of NF-κB expression in all mutants in the presence of TNF or IL-1β stimulation. These *in vitro* results correlated with patients’ data from PBMCs. Concerning the pro-inflammatory cytokines secretion, mainly IL-1β induced a significant and persistent enhancement of IL-6 and IL-8 in PBMCs carrying the S59P mutation.

**Conclusions:**

The novel S59P mutation leads to defective cellular trafficking and to constitutive activation of TNF-R1. This mutation also determines constitutive activation of the IL-1R pathway, inhibition of apoptosis and enhanced and persistent NF-κB activation and cytokine secretion in response to IL-1β stimulation.

**Electronic supplementary material:**

The online version of this article (doi:10.1186/s13075-015-0604-7) contains supplementary material, which is available to authorized users.

## Introduction

Tumor necrosis factor receptor-associated periodic syndrome (TRAPS; OMIM 142680) is the second most common inherited autosomal dominant autoinflammatory disease [1]. It is caused by mutations in the *TNFRSF1A* gene located on chromosome 12p13 and encodes the 55 kDa receptor for tumor necrosis factor receptor 1 (TNF-R1). The disease is clinically characterized by periodic fever, abdominal pain, skin lesions, conjunctivitis, myalgia and pericarditis. Disease onset generally occurs in early childhood but it can present in adults, and inflammatory attacks can last several weeks. The most severe complication is AA-type serum amyloidosis, which can lead to renal impairment and failure [2-4].

The TNF-R1 presents an extracellular domain involved in ligand binding, a transmembrane domain involved in receptor solubilization and an intracellular region where death domains (DD) are involved in signal transduction [5,6]. The binding of TNF to TNF-R1 causes recruitment of several intracellular adaptor proteins leading to downstream signaling events that activate the inflammatory (complex I) and apoptotic (complex II) processes. Complex I activation prevails and leads to NF-κB activation and transcription of anti-apoptotic and pro-inflammatory genes [7-27].

Almost all known mutations associated with TRAPS are characterized by the replacement of amino acids in the extracellular domain of the TNF-R1. More than 100 mutations are registered in the INFEVERS database [18], which are discerned in high-penetrance and low-penetrance mutations. The former affects mainly the cysteine residues in the extracellular cysteine-rich domains (CRDs) involved in disulfide bond formation and extracellular TNF-R1 folding and function. These mutations are associated with the most severe clinical phenotype, in which there is high risk of AA-type serum amyloidosis. Low-penetrance mutations include genetic variants that introduce or remove proline residues (such as P46L, L57P, S86P, and R92P) or affect hydrogen bond stabilization of the receptor (such as T50M) [19]. These mutations affect the secondary structure of TNF-R1 and are associated with a typical TRAPS phenotype, but in these cases the risk of AA-type serum amyloidosis is low [20-22]. Other low-penetrance *TNFRSF1A* variations, such as R92Q, are associated with milder clinical manifestations, suggesting that in these cases other genetic and/or environmental factors can play a role in the disease’s pathogenesis [20-26]. The high penetrance *TNFRSF1A* mutations involving the cysteine residues (C29F, C30R/S, C33G/Y, Y38C, C52F, C55S, C70R/Y, and C88R/Y) are less frequent (34%) than those with a low penetrance (76%), the latter being mainly represented by the T50M (15.8%) and R92Q (28.9%) variants [21].

We recently identified a novel mutation in the *TNFRSF1A* gene (exon 3, c.262 T > C) in an adult TRAPS patient. Clinically, the patient had fever, leukocytosis, recurrent bronchopneumonia, palpebral ptosis, episodes of arthralgia, myalgia and intermittent erythematosus skin lesions of the limbs and trunk. His serum immunoglobulin D (IgD), immunoglobulin A (IgA) and serum amyloid A protein (SAA) levels were elevated. While traditional therapy with the TNF inhibitor etanercept was unsuccessful in this patient, alternative therapy with the interleukin-1 receptor antagonist (IL-1Ra) anakinra promptly induced disease remission. The identified new genetic variant results in a proline for serine amino acid substitution (S59P or p.Ser88Pro) at residue 88, localized in the extracellular domain of the mature protein. This variant might be included in the above-described category of mutations that introduce proline residues. The distinctive cyclic structure of proline’s side chain gives it an exceptional conformational rigidity, interfering in peptide bond formation with other amino acids and affecting the secondary structure of the protein [27].

Overall, the molecular pathogenesis of TRAPS is not yet completely understood. The change in secondary structure might cause defective TNF-R1 trafficking, altered ligand binding affinity, reduced activation-induced shedding and impaired cell signaling. Based on these premises, several hypotheses have been formulated: the shedding hypothesis [1,28,29], the misfolding hypothesis [30-33], the NF-κB hypothesis [34,35] and the recent ROS hypothesis [36,37].

Since the patient carrying the S59P mutation was responsive to IL-1Ra but not to anti-TNF therapy, the aim of this study was to verify whether this mutation modifies the cell signaling, in particular in the NF-κB pathway, after TNF and IL-1β stimulation.

## Methods

### Wild-type and mutant *TNFRSF1A* cDNA vectors

We purchased the vector pCMV6-AC (OriGene Technologies, Inc. Rockville, MD, USA) containing wild-type (WT) *TNFRSF1A* cDNA ready for transfection. Three new constructs containing three mutant *TNFRSF1A* - cDNA (c.262 T > C (S59P); c.362G > A (R92Q) and c.236C > T (T50M)) were obtained using site-direct mutagenesis, and the following mutagenic primers were used: TNFRFS59P: 5′AGTGTGAGAGCGGC***C***CCTTCACCGCTTCAG3′; TNFRFR92Q: 5′ CTTCTTGCACAGTGGACC***A***GGACACCGTGTGTGGCTG 3′; TNFRRT50M: 5′ CTCACACTCCCTGCAGTCC***A***TATCCTGCCCCGGGCCTGG 3′.

All the products were sequenced along the full length of the *TNFRSF1A* coding region to ensure that only the desired mutations were introduced. Plasmid isolation from bacterial culture was carried out using the PureYield Plasmid Maxiprep System (Promega, Milano, Italy).

### Stable transfection of cell line HEK-293

Human embryonic kidney (HEK-293) cell line was used for the transfection. This cell line expressed moderate levels of endogenous TNF receptors and exhibited high transfection efficiency. A total of 4 μg of plasmid were incubated with 10 μL Lipofectamine™ 2000 (Invitrogen, San Giuliano Milanese, Italy) in 500 μL serum and antibiotic-free DMEM (Invitrogen, San Giuliano Milanese, Italy) for 20 minutes at 25°C. The reagent was then directly added to each cell culture well (200,000 cells) in a 6-well plate format. After 6 hours, media were replaced with fresh serum-supplemented media, and the cells were maintained at 37°C for the subsequent 24 hours. To obtain stable transformed cells, they were treated with 0.8 mg/mL Geneticin® (G418) (Invitrogen, San Giuliano Milanese, Italy) for 15 days.

### Patients

Two TRAPS patients were referred to the Rheumatology Unit of University of Padova and were studied: one carrying the S59P mutation and the other the R92Q mutation. Moreover, 5 subjects (3 males and 2 females, age range: 30 to 50 years) without any variants in the *TNFRSF1A* gene were studied as healthy controls. At the time the study was carried out, the TRAPS patients were on anakinra and infliximab therapy, respectively, and did not present inflammatory symptoms. The study protocol was approved by our institutional review board (Comitato Etico per la Sperimentazione, Azienda Ospedaliera di Padova, protocol number: 0060237) and the patients gave their fully informed written consent to participate in the study. The clinical characteristics of the enrolled TRAPS patients are detailed in Table 1.

**Table 1:** **Clinical characteristics of the two TRAPS patients included in the study**

	**TRAPS patients**	
*TNFRSF1A* variants	S59P	R92Q
Ethnicity/gender	Italian/Male	Italian/Female
Age at onset (year)	49 years (1991)	41 (2007)
Age at TRAPS diagnosis (year)	67 years (2009)	45 (2011)
Age at enrolment (year)	71 years (2013)	46 (2012)
Clinical manifestations at onset	Episodes of recurrent bronchopneumonia, fever, leukocytosis, refractory iron-deficiency anaemia, myalgia, intermittent erythematosus skin lesions on limbs and trunk and one episode of pericarditis.	Episodes of fever, myalgia, arthritis, headache and episcleritis.
Frequency of attacks (number per year)	4	6
Duration of the attacks (days)	7-14	7-14
Amyloid deposits	Yes (Spleen; 1996)	No
**Haematological and biochemical indices at diagnosis during an attack**		
Polymorphonuclear cells	24,190/μL	3,790/μL
Haemoglobin	10 g/L	12 g/L
Platelet count	842,000/μL	283,000/μL
Proteinuria	Absent	Absent
C-reactive protein	234 mg/L	20 mg/L
Serum amyloid A protein	1270 mg/L	59 mg/L
Serum IgD	271 g/L	44 g/L
Serum IgA	5.03 g/L	3.7 g/L
Erythrocyte sedimentation rate	120 mm/h	44 mm/h
Acute phase response	Persistent	Intermittent
Treatment	Prednisone 25 mg/day (1992–2008)	Sulfasalazine 2 g/day (since 2010)
	Prednisone 7.5 mg/day (2008–2011)	Infliximab 6 mg/kg once per month (2012–2014)
	Etanercept 25 mg twice per week (2011–2012)	Methotrexate 15 mg/week (since 2013)
	Anakinra 100 mg/day (since 2012)	Etanercept 25 mg twice per week (since 2014)
Response to treatment	Partial to prednisone	Partial
	Unresponsive to etanercept	
	Complete to anakinra	

According to our medical file, which includes 300 Italian patients with a clinical suspect of TRAPS, none carried the T50M mutation.

### *TNFRSF1A* mutation detection

Genomic DNA was extracted from whole blood using the standard method (MagNA Pure System, Roche S.p.A., Monza, Italy). After amplification by polymerase chain reaction with primers as described by D’Osualdo *et al*. [38], sequencing of the *TNFRSF1A* gene was performed using the automatic sequencer 3130ABI PRISM Genetic Analyzer (Applied Biosystems, CA, USA). Chromatograms were analyzed with Chromas Lite 2.01 software (Technelysium Pty Ltd., South Brisbane, QLD, Australia).

### Peripheral blood mononuclear cells isolation and culture

Blood (20 mL) was collected with EDTA K_3_ test tubes and was processed within 2 hours of sampling to isolate mononuclear cells by gradient centrifugation (Histopaque®-1077, Sigma-Aldrich, Milano, Italy). Peripheral blood mononuclear cells (PBMCs) were suspended in culture medium (Roswell Park Memorial Institute Medium (RPMI) supplemented with 0.1% gentamicin, 10% FCS and 1% glutamine; Invitrogen, San Giuliano Milanese, Italy) and incubated at 37°C for 24 and 72 hours prior to experimental manipulation to prevent any experimental anomalies arising as a consequence of therapy.

### Immunofluorescence

HEK-293 transfected with WT or mutant *TNFRSF1A* were seeded onto slides (3 × 10^5^) and maintained in culture for 24 hours. Cells were stimulated with or without TNF (60 ng/mL) (Sigma-Aldrich, Milano, Italy) for 1 hour to induce TNF receptor expression. They were then washed in PBS 1X and fixed in 4% formaldehyde fixative (Sigma-Aldrich, Milano, Italy) for 10 minutes at room temperature.

Dual indirect staining was performed using primary monoclonal mouse antibody anti-human CRI/TNF-R1-PE (R&D Systems Inc.) Minneapolis, Minnesota, USA and the appropriate Alexa Fluor 488-conjugated secondary antibody (Invitrogen, San Giuliano Milanese, Italy). The cells were washed in PBS 1X, incubated with NH_4_Cl (50 mM) for 10 minutes and with Triton X-100 (0.1%) for 5 minutes. The cells were then washed in PBS 1X and incubated with PBS with BSA (1%) for 45 minutes. The first primary antibody was added to the cells and incubated for one hour at room temperature in the dark. Cells were then washed three to four times in PBS 1X. The secondary antibody was added to the cells and incubated for one hour at room temperature in the dark. Confocal microscopy was performed using a Radiance 2000 confocal laser scanning microscope (Bio-Rad Laboratories Inc., Milano, Italy).

### Reverse phase protein array analysis

Reverse phase protein array (RPPA) analysis was performed using a procedure previously optimized and validated [39]. Briefly, cell lysates were solubilized in 4× SDS loading buffer (Sigma-Aldrich, Milano, Italy) and heated for 5 minutes at 95°C. Samples were spotted in duplicate onto nitrocellulose-coated glass slides (Grace Bio-labs Bend, Oregon, USA) using a microarraying robot (MicroGrid 610, Digilab, Marlborough, MA, USA). The printed slides were blocked and incubated overnight at 4°C with shaking with the specific primary antibodies (Cell Signaling Technology, Danvers, MA, USA). Additional file 1 shows in more detail the list of primary antibodies used for RPPA analysis with their respective signaling pathways. β-actin was included as a housekeeping protein to control protein loading. After incubation with infrared secondary antibodies (800 CW LI-COR anti-rabbit antibody and 700 CW LI-COR anti-mouse antibody) Lincoln, Nebraska, USA, the slides were scanned with a Licor Odyssey scanner (LI-COR, Biosciences Lincoln, Nebraska, USA) at 21 μm resolution at 700 and 800 nm. The fluorescent data were processed with GenePix Pro-6 Microarray Image Analysis software (Molecular Services Inc. Sunnyvale, California, USA). Protein signals were determined with background subtraction and normalization to the internal housekeeping targets using an Reverse-phase protein analyzer [40].

### Immunoblotting

WT or mutant *TNFRSF1A* HEK-293 and PBMCs were seeded (5 × 10^6^) onto Petri dishes (diameter: 10 cm) and maintained in a laboratory culture for 24 hours. Cells were incubated with and without TNF (6 ng/mL) or with IL-1β (1 ng/mL) (Sigma-Aldrich, Milano, Italy) for 10 minutes to activate signaling pathways. Following stimulation, the Petri dishes were immediately transferred to an ice bath, and the cells were washed twice with cold PBS, resuspended in 100 μL of cold lysis buffer (20 mM Tris–HCl, pH 7.5, 150 mM NaCl, 1 mM EDTA, 1% Triton X-100, 50 mM NaF, 10 mM Na_4_P_2_O_7_, 1 mM Na_3_VO_4_, and 10% protease inhibitor cocktail (Sigma-Aldrich, Milano, Italy)). Lysates were centrifuged for 10 minutes at 14,000 rpm at 4°C and the supernatants were collected. The pellets were resuspended in a nuclear extraction buffer mix (Thermo Scientific Inc., Rockford, USA) and proceeded to generate nuclear extracts, as described in the manufacturer’s instructions. Total cytosolic and nuclear proteins were measured using the Bio-Rad Protein Assay Kit (Bio-Rad Laboratories, Milano, Italy).

For each sample, 40 μg proteins were electrophoresed using a 4-12% NuPAGE® Novex Bis-Tris SDS-PAGE Gel or 3-8% NuPAGE® Novex Tris-Acetate SDS-PAGE Gel (Invitrogen Life Technologies, Monza, Italy), and were electrophoretically transferred to nitrocellulose membrane (iBlot® Transfer Stack, Invitrogen Life Technologies, Monza, Italy) using iBlot® Dry Blotting System (Invitrogen Life Technologies, Monza, Italy). The membranes were incubated for 1 hour in blocking buffer (5% low-fat powder milk resuspended in PBS-T (PBS with 0.1% Tween-20)). They were then incubated overnight with the primary antibodies (anti-phospho IkB-α (Ser^32^) and anti-phospho-NF-κB subunit p65 (Ser^536^) (Cell Signaling Technology, Danvers, MA) and anti-β-actin (Sigma-Aldrich, Milano, Italy)) diluted 1:5,000 (β-actin) or 1:3,000 (all the others) in blocking buffer. The blots were washed three times in PBS-T for 15 minutes and incubated for 1 hour with alkaline phosphatase-conjugated anti-rabbit or anti-mouse secondary antibodies (Cell Signaling Technology, Danvers, MA, USA). The blots were washed three times in PBS-T for 15 minutes and developed with the ECL Advance Western Blot Detection Kit (GE Healthcare Technologies, Milano, Italy).

### Cytokine assay

Cells transfected with WT or mutant *TNFRSF1A* and PBMCs cultured for 24 and 72 hours were incubated at 200 × 10^3^/mL in 24-well plates in culture medium. The cells were stimulated with and without TNF (6 ng/mL) or IL-1β (1 ng/mL) or lipopolysaccharide ((LPS) 1 μg/mL) as positive controls (PC) for four hours. Supernatants were collected, centrifuged to remove cells and debris and stored at −80°C. IL-1β, IL-6, IL-8 and TNF cytokine analysis was performed using an immunometric assay (IMMULITE-Siemens, Milano, Italy).

### Statistical analysis

Two sets of data were analyzed using an independent sample t test. The data of more than two groups were analyzed using one-way analysis of variance (ANOVA) and Bonferroni’s test for pairwise comparisons. The statistical software Package for the Social Sciences (SPSS version 9) was used. All experimental data were obtained from a series of independent experiments (n = 3).

## Results

### S59P is a novel TRAPS-associated mutation

**Figure 1:**
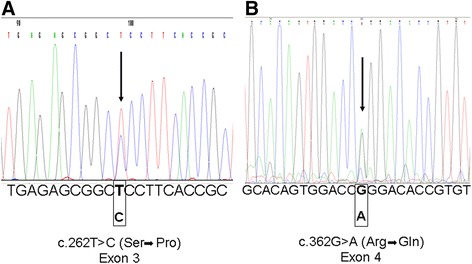
DNA sequence electropherograms of *TNFRSF1A*. **A**: electropherogram of the heterozygous single-base mutation (c.262 T > C) in exon 3, resulting in a Pro for Ser amino acid substitution. **B**: electropherogram of the heterozygous single-base mutation (c.362G > A) in exon 4, resulting in an Arg for Gln amino acid substitution.

Based on the clinical suspect of TRAPS, a 67-year-old Italian male (clinical characteristics detailed in Table 1) was sent to our department. By performing *TNFRSF1A* sequencing, this patient was demonstrated to carry in heterozygosis a mutation which has never been previously described (Figure 1, panel A). This single-base mutation (c.262 T > C), located in exon 3, results in a proline for serine amino acid substitution (S59P) at residue 88 of the mature protein. We studied another TRAPS patient (clinical characteristics also detailed in Table 1) who carried in heterozygosis the common single-base mutation (c.362G > A) in exon 4 (Figure 1, panel B), resulting in an arginine for glutamine amino acid substitution (R92Q) at residue 121 of the mature protein.

### Different *TNFRSF1A* mutants activate specific TNF-R1 downstream signaling pathways

To ascertain whether the novel S59P mutation is causative of an altered TNF-R1 expression and function, *in vitro* experiments were performed using the HEK-293 cell line transfected with a plasmid expression vector carrying the S59P-mutated *TNFRSF1A.* For comparison, the same HEK-293 cells were transfected with the WT *TNFRSF1A* and the R92Q and T50M *TNFRSF1A* mutants.

**Figure 2:**
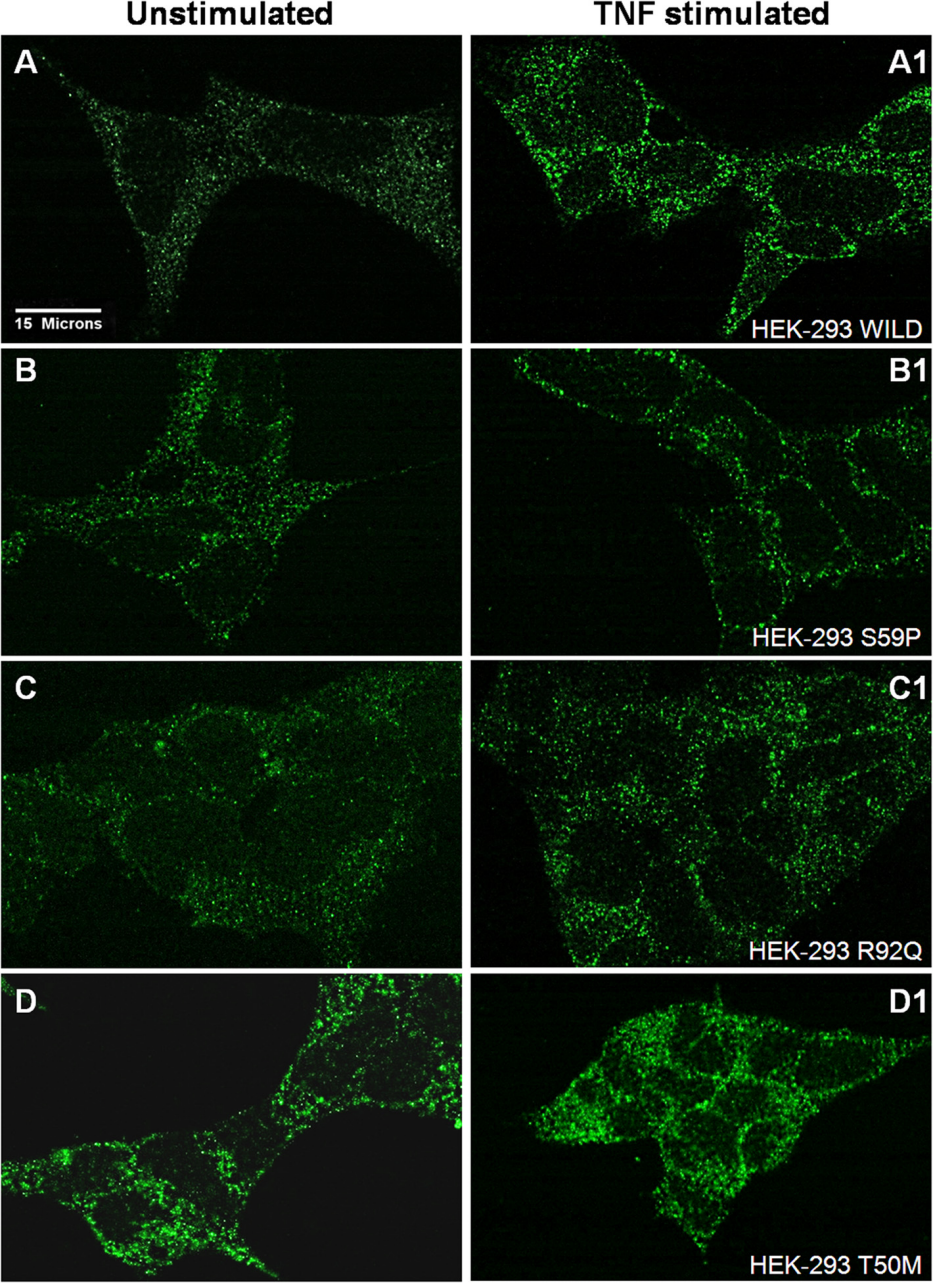
TNF-R1 expression in cell lines. Confocal microscopy analysis of anti-TNF-R1 (green) immunofluorescent staining. HEK-293 cells transfected with wild-type (WT) or mutant *TNFRSF1A* remained unstimulated or they were stimulated with 60 ng/mL TNF. **Panel A** (without TNF) and **A1** (with TNF) are WT *TNFRSF1A* HEK-293. **Panel B** (without TNF) and **B1** (with TNF) are S59P *TNFRSF1A* HEK-293. **Panel C** (without TNF) and **C1** (with TNF) are R92Q *TNFRSF1A* HEK-293. **Panel D** (without TNF) and **D1** (with TNF) are T50M *TNFRSF1A* HEK-293.

We first analyzed by indirect immunofluorescence TNF-R1 expression in unstimulated (Figure 2, left panels) and TNF-stimulated (Figure 2, right panels) WT, S59P, R92Q and T50M HEK-293 transfected cells. In unstimulated WT cells (Figure 2A), the TNF-R1 was weakly expressed in the cytosol. A similar pattern was observed in S59P (Figure 2B) and R92Q (Figure 2C) HEK-293 mutant cells, while in the presence of T50M mutation a more intense cytosolic fluorescence with a granular-type pattern could be identified (Figure 2D). TNF at the dosage of 60 ng/mL caused an enhanced expression and granularity of cytosolic TNF-R1 not only in WT (Figure 2A1), but also in all mutant HEK-293 cells. Although a clear differentiation of cell surface from intracellular localizations was not possible, an enforced fluorescence with granular pattern outlining cell shape was observed in all TNF-stimulated cells.

**Figure 3:**
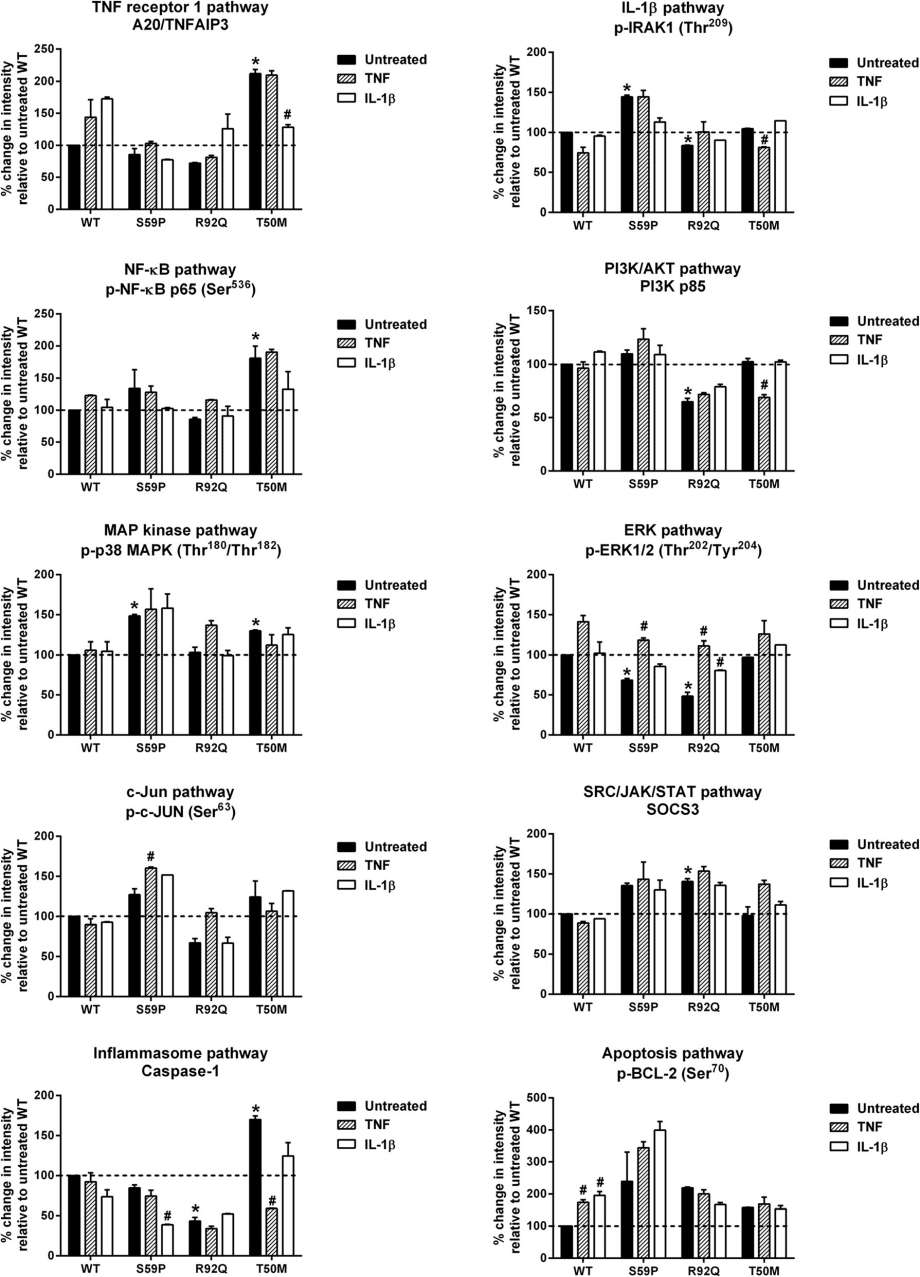
Reverse phase protein array (RPPA) results. In each panel the percentage changes in fluorescence intensity relative to untreated wild-type (WT) HEK-293 cells are shown. For any signaling pathway, a key component molecule is shown. Bonferroni’s test for pairwise comparisons: *P <0.05 with respect to untreated WT HEK-293 cells; #*P* <0.05 with respect to its own untreated control.

We then verified whether the *TNFRSF1A* mutations are associated with a constitutive activation of the TNF-R1 or other signaling pathways, and whether these mutations modify intracellular signaling in response to TNF and IL-1β, taking into account that the patient carrying the S59P mutation had a complete clinical response when treated with IL-1Ra but not with anti-TNF therapy. RPPA with unstimulated and TNF- or IL-1β-stimulated cells were performed and the following pathways analyzed: TNF-R1, MAP kinase, c-Jun, IL-1β, NF-κB, ERK, inflammasome, PI3/AKT, SRC/JAK/STAT and apoptosis. In Figure 3 the percentage changes in fluorescence intensity relative to untreated WT HEK-293 cells are shown; for any studied signaling pathway, a key component molecule is shown. Additional file 2 shows in more detail all remaining RPPA data for any studied signaling pathway. A constitutive activation of the TNF-R1 and inflammasome signaling pathways, characterized by increased A20/TNFAIP3, p-NF-κB and caspase 1, was associated with the T50M, not with the S59P or R92Q mutations. The newly identified S59P mutation was associated with the constitutive activation of IL-1β (pIRAK), MAPK (p38MAPK) and SRC/JAK/STAT3 (SOCS3) pathways. In R92Q mutants, the constitutive activation of SRC/JAK/STAT3 was associated with the inhibition of PI3K/AKT, ERK, c-Jun and inflammasome pathways. Apoptosis (BCL-2) was constitutively inhibited in all mutants. TNF and IL-1β activated the TNF receptor pathway and inhibited apoptosis in WT cells. The TNF-R1 pathway was not activated by TNF or IL-1β in mutant cells, nor was it even inhibited by IL-1β in the T50M mutant. Differently from the R92Q and T50M mutants, the S59P mutation did not abolish the anti-apoptotic effects of TNF and IL-1β.

### *TNFRSF1A* mutants activate NF-κB pathway in transfected cells and in peripheral blood mononuclear cells

We then ascertained in more depth whether a different response to TNF and IL-1β in terms of activation or inhibition of the NF-κB pathway, depends on *TNFRSF1A* mutations. This objective took into consideration that NF-κB is one of the main TNF-R1 downstream signaling pathways [41], and that both pathways were differently affected by S59P, R92Q and T50M mutations (shown in RPPA results). The effects of TNF and IL-1β on two key NF-κB signaling molecules, p-IκB-α (Ser^32^) and p65 component (p-NF-κB (Ser^536^)), were assessed by Western blot analysis in WT, S59P, R92Q and T50M HEK-293, and in PBMCs obtained from TRAPS patients and controls.

**Figure 4:**
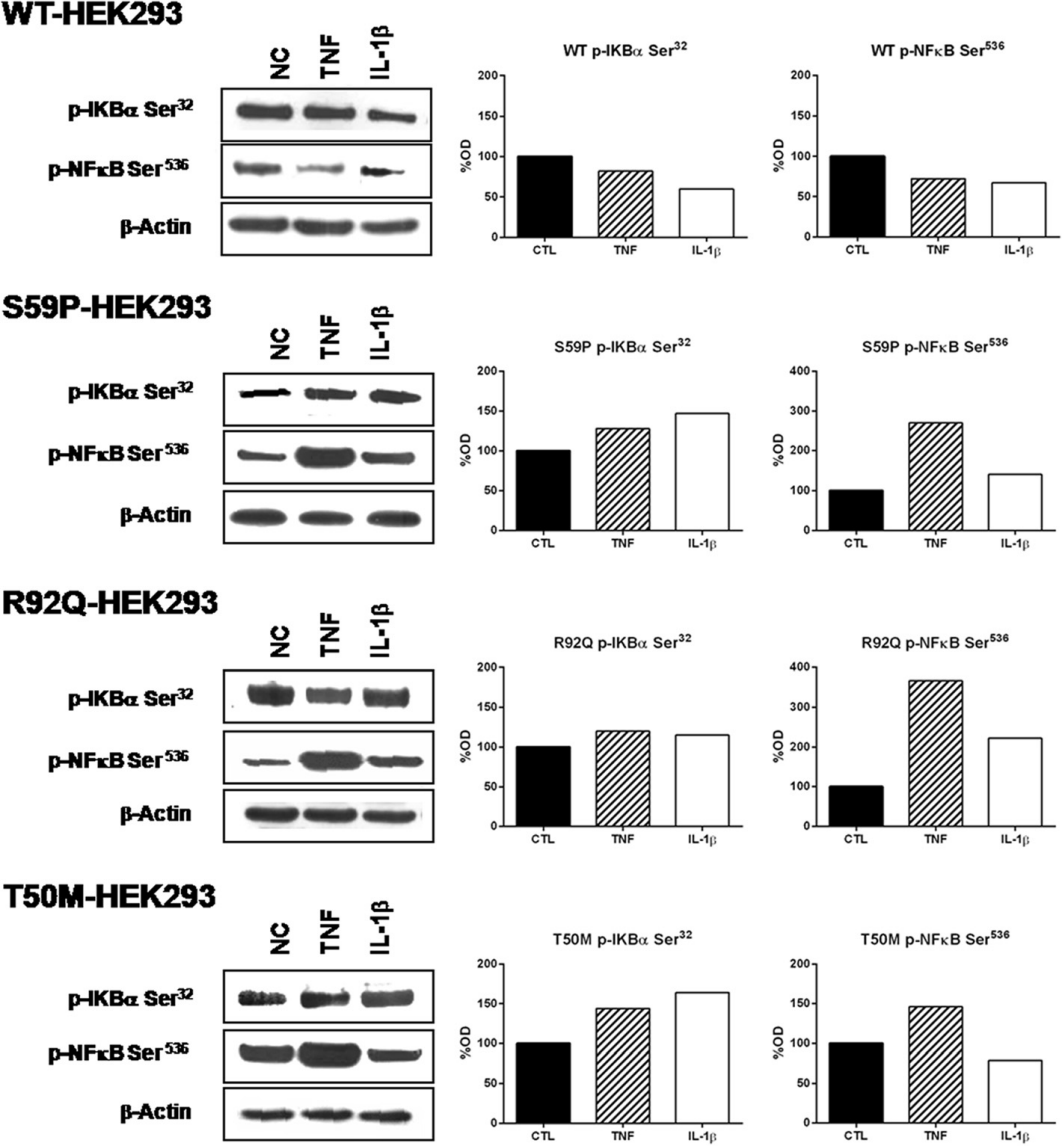
Effects of TNF and IL-1β on the NF-κB pathway in cell lines. Wild-type (WT) and mutant *TNFRSF1A* HEK-293 unstimulated (NC) or stimulated for 10 minutes or with TNF (6 ng/mL) or IL-1β (1 ng/mL). Western blot shows p-IκBα (Ser^32^) and p65 subunit of NF-κB (Ser^536^) and the corresponding β-actin, used as a control. The histograms show semi-quantification of band intensities after normalization against the negative control (100%) (OD; Image J software, version 1.47 NIH, Bethesda, Maryland, USA). Columns indicate percent values.

Figure 4 shows Western blot results of transfected HEK-293 cell lines. TNF and IL-1β reduced the phosphorylation of IκB-α (p-IκB-α Ser^32^) in WT cells, while the opposite was found when they were added to S59P and T50M mutant cells. In R92Q cells, little if any variation in IκB-α phosphorylation was seen. The Western blot analysis of the p65 component in isolated nuclei (p-NF-κB Ser^536^) showed that TNF and IL-1β did not activate p65 in WT HEK-293 cells. By contrast, this component was phosphorylated by TNF in all mutants, and by IL-1β in S59P and R92Q, but not T50M mutants. Confirming RPPA findings, p65 was constitutively activated in the T50M HEK-293, and this activation was sustained by TNF and partly counteracted by IL-1β.

**Figure 5:**
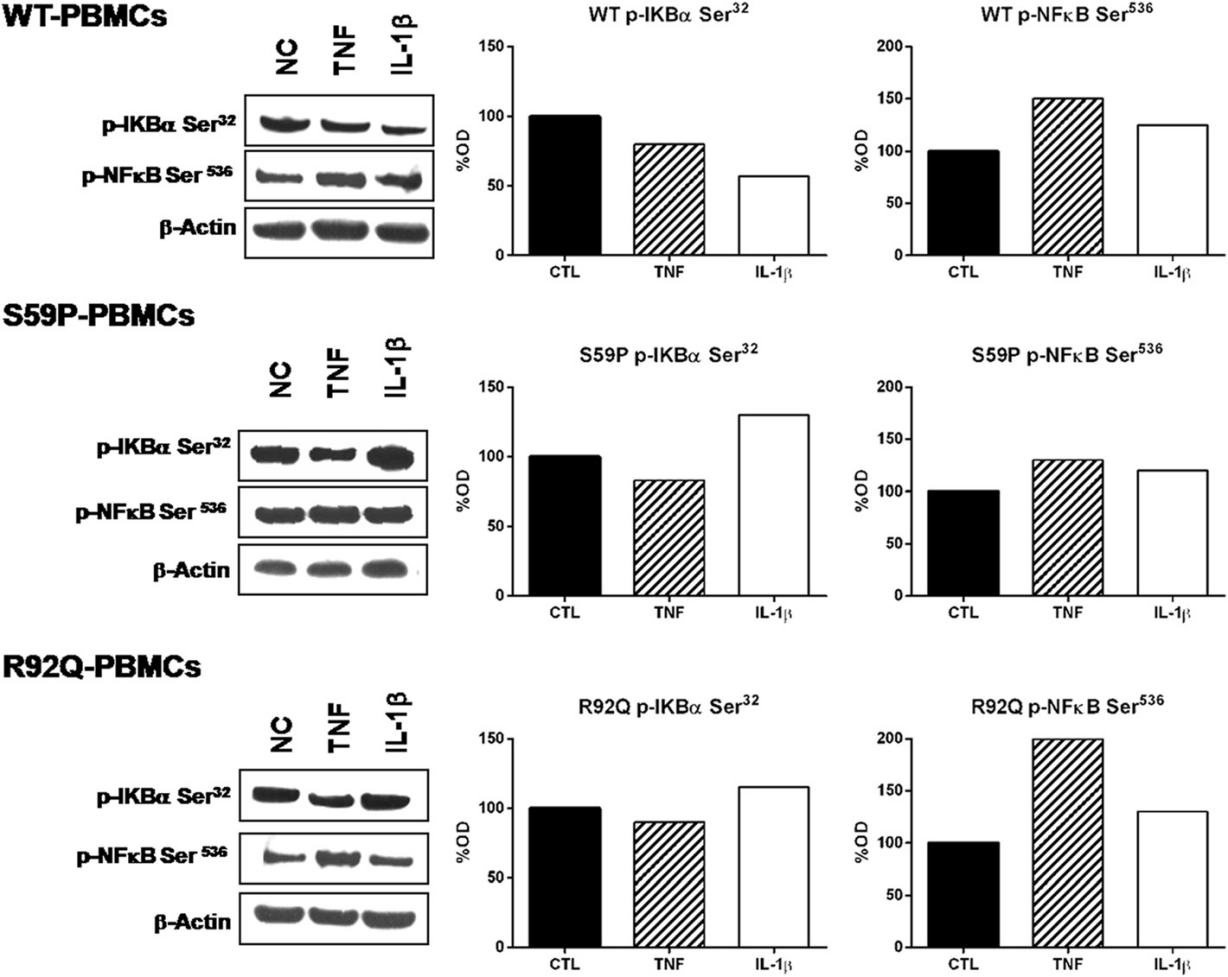
NF-κB pathway in peripheral blood mononuclear cells. Control (wild-type (WT)) and TRAPS peripheral blood mononuclear cells unstimulated or stimulated for 10 minutes with TNF (6 ng/mL) or IL-1β (1 ng/mL). Western blot shows p-IκBα (Ser^32^) and p65 subunit of NF-κB (Ser^536^) and the corresponding β-actin, used as control. The histograms show semi-quantification of band intensities after normalization against the negative control (100%) (OD; Image J software, version 1.47 NIH, Bethesda, Maryland, USA). Columns indicate percent values.

Figure 5 shows Western blot analysis of p-IκB-α (p-IκB-α Ser^32^) and of nuclear p65 component (p-NF-κB Ser^536^) in control and TRAPS PBMCs kept in culture for 24 hours unstimulated or stimulated with TNF and IL-1β. In basal conditions IκB-α phosphorylation was higher in TRAPS with respect to control PBMCs. In both control and TRAPS patients TNF reduced IκB-α phosphorylation. IL-1b reduced the phosphorylation of IkB-a in control PBMCs, while enhnacing it in TRAPS PBMCs. In the same experimental conditions, p65 activity in isolated nuclei was constitutively elevated only in S59P TRAPS PBMCs. Both TNF and IL-1β induced the p65 phosphorylation in controls and TRAPS PBMCs.

### *TNFRSF1A* mutants increase pro-inflammatory cytokine secretion in transfected cells and peripheral blood mononuclear cells

We then examined if NF-κB activation might be functionally linked to the secretion of inflammatory cytokines. Levels of IL-8 were measured in WT, S59P, R92Q and T50M HEK-293 supernatants. Levels of IL-1β, IL-6, IL-8 and TNF were measured in PBMCs supernatants. Cells were incubated in fresh media with or without TNF (6 ng/mL), IL-1β (1 ng/mL) or LPS (1 μg/mL) as a positive control for 4 hours.

**Figure 6:**
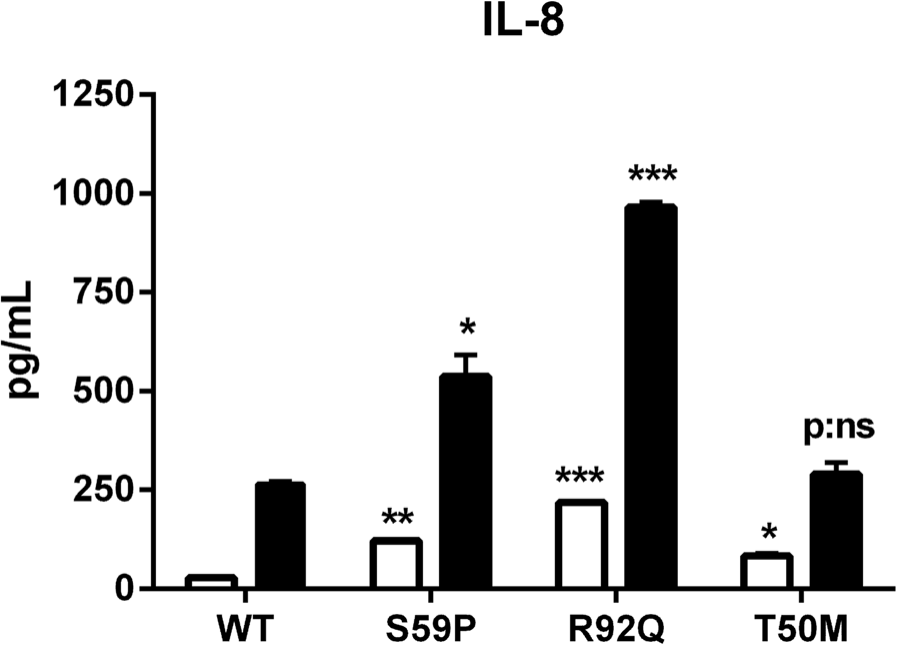
Proinflammatory cytokine response in cell lines. IL-8 levels in wild-type (WT) and mutant *TNFRSF1A* HEK-293 unstimulated (white columns) or stimulated (black columns) for 4 hours at 37°C with TNF (6 ng/mL). Data displayed as mean ± SEM. *P <0.005; **P <0.001; ***P <0.0001 respect to WT.

Non-stimulated S59P, R92Q and T50M HEK-293 released higher levels of IL-8 compared to WT cells (t = −29, *P* <0.0001; t = −89.2, *P* <0.0001; t = −8.6, *P* <0.001) (Figure 6). TNF induced a significantly higher IL-8 secretion in S59P (t = −5.1, *P* <0.005) and R92Q (t = −52.3, *P* <0.0001), not in T50M (t = −0.8; p: not significant) mutants, with respect to WT HEK-293 cells.

**Figure 7:**
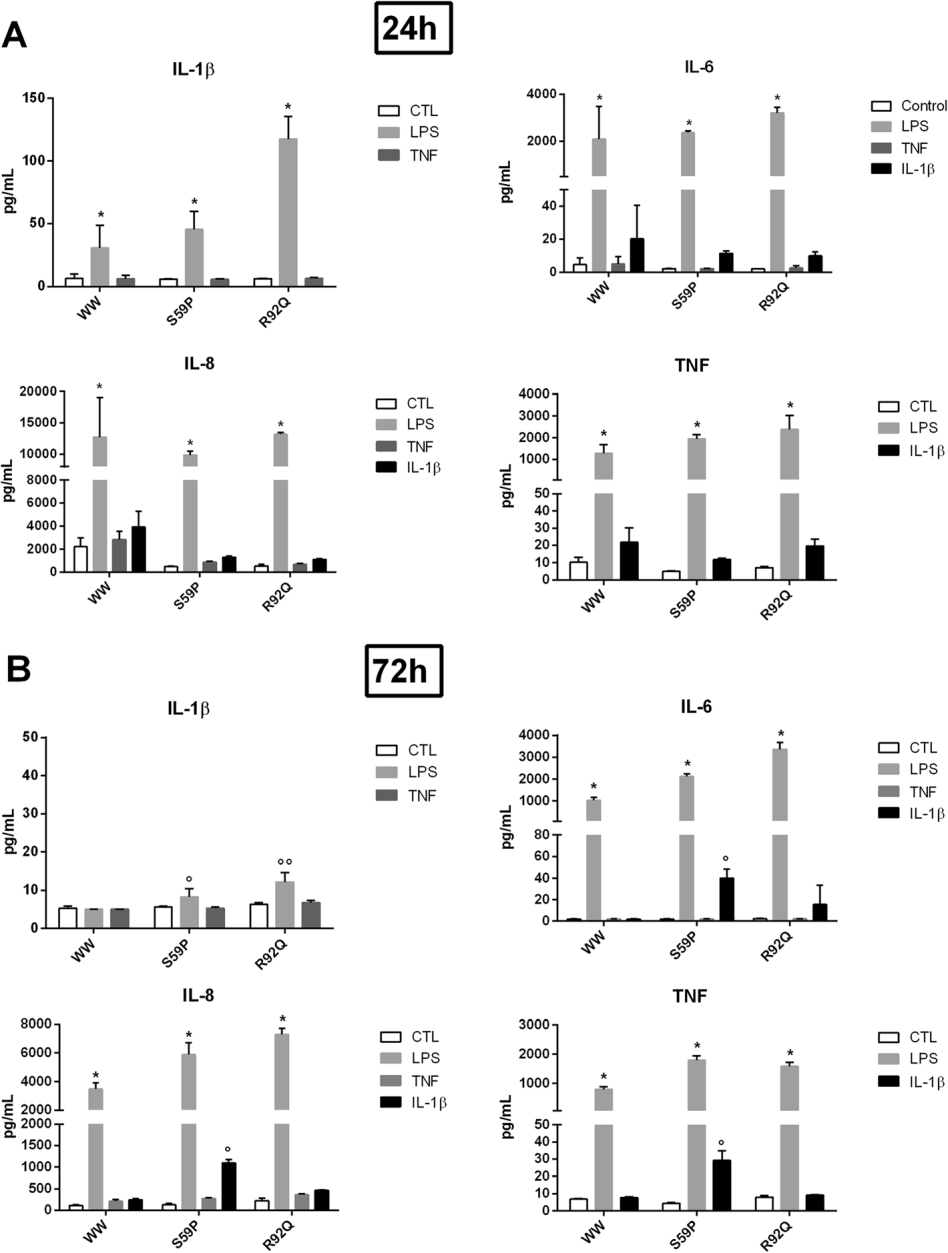
Proinflammatory cytokine response in peripheral blood mononuclear cells. Cytokine levels (IL-1β, IL-6, IL-8 and TNF) in control (WW) and TRAPS peripheral blood mononuclear cells maintained in culture for 24 **(panel A)** and 72 hours **(panel B)** and unstimulated or stimulated for 4 hours with LPS (1 μg/mL) (positive control), TNF (6 ng/mL) or IL-1β (1 ng/mL). Columns represent mean values ± SEM. *P <0.0001; °*P* <0.05; °°*P* <0.001 with respect to WW.

In all PBMCs (Figure 7, upper panels) a 24-hour culture of LPS induced significantly higher IL-1β (control: F = 35, *P* <0.0001; S59P: F = 31, *P* <0.0001; R92Q: F = 153, *P* <0.0001), IL-6 (control: F = 44, *P* <0.0001; S59P: F = 4,046, *P* <0.0001; R92Q: F = 661, *P* <0.0001), IL-8 (control: F = 45, *P* <0.0001; S59P: F = 808, *P* <0.0001; R92Q: F = 5,755, *P* <0.0001) and TNF (control: F = 198, *P* <0.0001; S59P: F = 448, *P* <0.0001; R92Q: F = 53, *P* <0.0001) secretion. Both TNF and IL-1β induced significantly higher IL-8 release in the control with respect to the TRAPS PBMCs (TNF: F = 29, *P* <0.0001; IL-1β: F = 15, *P* <0.0001).

In a 72-hour culture (Figure 7, lower panels), LPS no longer affected IL-1β secretion in control PBMCs, but it continued to induce IL-1β secretion in the S59P (F = 7.55, *P* = 0.012) and R92Q (F = 20.8, *P* <0.0001) TRAPS PBMCs. LPS maintained its stimulatory effect at 72 hours on IL-6 (control: F = 179, *P* <0.0001; S59P: F = 1055, *P* <0.0001; R92Q: F = 489, *P* <0.0001), IL-8 (control: F = 224, *P* <0.0001; S59P: F = 167, *P* <0.0001; R92Q: F = 1042, *P* <0.0001) and TNF (control: F = 323, *P* <0.0001; S59P: F = 507, *P* <0.0001; R92Q: F = 466, *P* <0.0001) secretion. TNF-stimulated IL-8 secretion was significantly higher in the S59P and R92Q TRAPS than in the control PBMCs (F = 24, *P* <0.0001). IL-1β stimulus maintained both IL-6 and IL-8 secretion higher in the S59P and R92Q TRAPS than in the control PBMCs (F = 11, *P* <0.005; F = 322, *P* <0.0001) (Figure 7, lower panels).

## Discussion

Mutations in the *TNFRSF1A* gene cause the autosomal-dominant, with incomplete penetrance, autoinflammatory disorder TRAPS. Mutations located in the CRDs region of the receptor are highly penetrant and associated with the most severe clinical phenotypes. However, these mutations are less frequent than those involving extracellular non-cysteine residues, with R92Q and T50M being the most common [1]. The cellular mechanisms underlying the pathophysiology of TRAPS are complex and depend on the mutation involved, which then defines some of its clinical features [21,42].

We identified a novel *TNFRSF1A* mutation in an adult patient diagnosed as having TRAPS. This patient had a variety of clinical manifestations, some of which were commonly associated with TRAPS (recurrent episodes of fever, myalgia and erythematosus skin lesions), and others which are reported less frequently (pericarditis and pneumonia) [43]. Sequencing of the *TNFRSF1A* gene was performed in this patient after about 18 years of recurrent episodes of bronchopneumonia, fever and other systemic signs of inflammation with an incomplete response to steroid treatment. This long lag time between disease onset and diagnosis depends on the lag time between disease onset and TRAPS first description [1]. The identified novel mutation was located in exon 3 and leads to the substitution of proline for serine at residue 88 of TNF-R1 (S59P).

Any amino acid substitution in the ectodomain of TNF-R1 might affect its physiology, including membranal expression levels, three-dimensional receptor folding and stimulus-dependent or independent activation. The novel S59P mutation determines the presence in the TNF-R1 of one more proline residue, which might interfere with three-dimensional receptor folding, since the distinctive cyclic structure of proline’s side chain gives it an exceptional conformational rigidity and might cause a bend in the receptor’s secondary amino acid structure [27]. These structural consequences might subsequently cause an aberrant behaviour of the receptor, including ligand independent gain-of-function, which underlies TRAPS pathophysiology.

To understand the functional features of this novel S59P mutation, we set out to compare it with two frequent mutations that are well known in the literature: the R92Q and T50M mutations [20-23,31-33,38,44,45]. These two mutations were chosen in view of several considerations: neither involves cysteine residues, the R92Q mutation is associated with a variable phenotype [23], and the T50M mutation is associated with a typical TRAPS phenotype [21].

As it is difficult to study the pathophysiologic mechanisms of TRAPS in the patients themselves, we employed an *in vitro* model and only the noteworthy results were examined using the patients’ cells. We first focused on the expression of TNF-R1 by immunofluorescence and found that this receptor is homogeneously distributed in the cytosol of WT cells. S59P and R92Q mutations did not significantly modify this pattern, while T50M caused an enhanced cytosolic TNF-R1 expression with a granular appearance. These results are supported by previous data from Rebelo *et al*. [32], who showed by flow cytometry that the WT and R92Q TNF-R1 have a significant cell surface expression, with a proportion being intracellular, whereas the T50M is almost completely retained in the cytoplasm. Based on the pattern of S59P expression, we might suggest that this novel mutation, similarly to R92Q and differently from T50M, does not promote intracellular retention of TNF-R1.

We then verified whether the exposure of cells to the TNF-R1 cognate ligand TNF exerts a mutation-dependent effect on receptor expression. In WT, S59P and R92Q mutant cells, TNF caused an enforced expression of the TNF-R1 mainly in the cytosol but also at the cell membrane, while in T50M mutant cells TNF induced an enforced expression of cytoplasmic aggregates. It might be suggested that S59P, similarly to the R92Q mutation, does not alter TNF-R1 trafficking from the cytoplasm to the surface membrane, while the T50M mutation causes a misfolded protein that overloads, leading to the formation of aggregates in the cytosol [32,45].

The pivotal role of an altered TNF receptor in TRAPS pathophysiology supports traditional therapy with etanercept, a fusion molecule of TNF-R2/FcIg [21,25,36], or with other anti-TNF agents such as infliximab, a monoclonal anti-TNF antibody; however, in most cases these therapies were shown to be only partially efficacious [46,47]. Therapy with anakinra, a recombinant interleukin-1 receptor antagonist, has recently proven to be a promising therapeutic alternative, preventing short-term disease relapse even in etanercept-resistant patients [33,48-50]. The success of this therapeutic approach, also recorded in our S59P TRAPS patient, might be due to increased IL-1β secretion for impaired autophagy [51], but also to the interactions occurring between IL-1 and TNF-R1 signaling [52]. TNF-R1 trimerizes before binding to TNF trimer, which induces TRADD-dependent and independent activation of several downstream signaling pathways, including the NF-κB, apoptosis, JNK, c-Jun, p38-MAPK and ERK [53]. IL-1β signaling is initiated with recruitment of MyD88 to the Toll-IL-1 receptor domain, followed by the phosphorylation of several kinases and activation of the NF-κB signaling pathway [49].

By studying all the above TNF-R1 signaling pathways and the IL-1 pathway in WT and mutant cells with a comprehensive RPPA approach, we demonstrated that in the presence of T50M, not S59P or R92Q mutations, TNF-R1, NF-κB and caspase 1 are constitutively activated. In agreement with the hypothesis that a possible association between TNF-R1 mutation and IL-1 signaling exists, constitutively activated IRAK and TAK proteins, involved in the IL-1 signaling pathway, were observed in cells carrying the S59P mutation. In R92Q cells we observed an attenuation of NF-κB, c-Jun, ERK, Akt, and inflammasome pathways and an increase in suppressor of cytokine signaling (SOCS) protein 3, which attenuates the intensity and/or duration of a cell’s response to a diverse range of extracellular stimuli by suppressing the signal transduction processes. These findings indicate that any *TNFRSF1A* mutant differently affects the pro-inflammatory signaling pathways downstream of TNF-R1: the T50M mutant activates TNF-R1 pro-inflammatory signaling pathways, the novel identified S59P mutant activates IL-1 signaling and the R92Q mutant inhibits, not activates, TRADD-dependent signaling. A common feature of all studied *TNFRSF1A* mutants was the constitutive inhibition of apoptosis by increased inhibition of *BCL-2* and *BAD*. Considering that the molecular pathogenetic mechanisms proposed for TRAPS include defective autophagy, ROS production, defective apoptosis and enhanced activation of NF-κB, our results suggest that a defective apoptosis is a common feature of different *TNFRSF1A* mutations, and that their differences in causing more or less severe forms of disease are probably related to their ability in sustaining ligand-dependent and independent NF-κB activation and inflammation.

For this reason the NF-κB pathway was studied in more detail in our experimental setting. The activated TNF receptor triggers a set of cascade events, including IKK complex activation, phosphorylation and IκB-α degradation, and activation and nuclear translocation of the NF-κB complex that in turn modulates the expression of a sequence of pro-inflammatory genes [10-17,26]. By RPPA analysis, NF-κB was shown to be activated both constitutively and after exposure to TNF and IL-1β in the T50M mutant. Western blot analysis confirmed that TNF and IL-1β induced the phosphorylation of IκB-α, which was higher and more persistent in T50M and S59P than in the WT *TNFRSF1A* cells. This phenomenon could be explained by the combined TNF-independent autoactivation of the mutated receptor and by the TNF-dependent activation of the WT receptor, since both receptors are present in these heterozygous cells. The IκB-α downstream signaling involves the p65 subunit, which plays an important role in protecting from apoptosis and innate immunity [13,54]. Confirming reports by Yousaf *et al*. on T50K [30] and of Churchman *et al*. on T50M [55], we found that the p65 subunit was constitutively active in the T50M *TNFRSF1A* cells and, using Western blot analysis (see Figure 4), we found that TNF induced a higher phosphorylation of the nuclear p65 subunit in the mutated rather than the WT *TNFRSF1A* cells. The constitutive and stimulus-dependent accumulation of p65 in the nucleus might confer insensitivity to apoptosis and keep the cells in a hyper-inflammatory state. Findings in an *in vitro* model correlated with results obtained from the PBMCs of TRAPS patients. The NF-κB pathway was constitutively activated and upregulated by inflammatory stimuli in the presence of TRAPS-associated mutations. IL-1β supported the significance of IκB-α phosphorylation and NF-κB activation in S59P TRAPS PBMCs.

NF-κB activation leads to pro-inflammatory cytokine secretion [11,13,14]. We investigated this phenomenon by measuring IL-8 in transfected cells and IL-8, IL-6, TNF and IL-1β in PBMCs from the S59P and R92Q TRAPS patients. A limitation of our study is that there was no patient with the T50M mutation because it was never found in our series of TRAPS patients, and this prevents from comparing accurately the results obtained with the other variants. TNF induced a significant increased release of IL-8 in R92Q and in S59P *TNFRSF1A* cells. This finding is in agreement with results described by Nedjai *et al*. [35] and was supported by the clinical data: IL-1β induced a significantly higher and persistent IL-6 and IL-8 secretion in the S59P TRAPS PBMCs and IL-6 secretion in the R92Q TRAPS PBMCs, which were maintained in long-term culture (up to 72 hours) in order to exclude any interference as a consequence of therapy.

IL-8 response to TNF stimulation of T50M cells was similar to WT cells in agreement with findings from Churchman *et al*. [55], who found that the PBMCs carrying the T50M or C88R mutation, despite having elevated basal p65 nuclear expression, did not show further increase in p65 or p50 NF-κB subunits following stimulation with TNF. This relative low sensitivity of T50M mutant cells to TNF might be due to the low membranal expression of TNF-R1, as previously shown by flow cytometric FACS analysis [55] and confirmed in the present study by immunofluorescence (see Figure 1). However, our findings are in disagreement with those from Nedjai *et al*. [35], who studied the T50K mutation. This supports the notion that the molecular structure of the replacement amino acid (Methionine-M not Lysine-K), although involving the same tyrosine residue at position 79 in the mature protein, might differently affect receptor function.

In view of these considerations, we performed the same experiments using IL-1β stimulation. In a physiological state, IL-1β did not apparently influence NF-κB activity in the cell line considered. Instead, in the *TNFRSF1A* mutated cells, this cytokine induced an inflammatory response. The IL-1R pathway proceeded in canonical NF-κB pathway. We suggest that IL-1b enhances the inflammatory response of TNRSF1A mutated cells due to their hyper-inflammatory background.

## Conclusions

We identified and functionally characterized a new (S59P) *TNFRSF1A* mutation. This mutation is associated with a TRAPS phenotype characterized by a high degree of inflammation only partially responsive to steroid treatment. This phenotype appeared to be highly responsive to anakinra, which was used in the patient in question over the course of one year. The S59P mutation determines a constitutive activation of the IL-1R pathway, inhibition of apoptosis, and an enhanced and persistent NF-κB activation and cytokines secretion in response to IL-1β stimulation.

## Additional files

Additional file 1:
**List of primary antibodies used in TNF-R1 signalling study. For lysate microarray (RPPA) analysis, they were diluted as shown in the table.**
Additional file 2:
**RPPA data obtained from unstimulated, TNF and IL-1β stimulated wild-type (WT) and mutant HEK-293 cells.** For any target, data are reported as mean ± SE (two independent experiments) percentage changes in fluorescence intensity, with respect of WT-HEK293 unstimulated cells. The statistical analysis was made by one-way analysis of variance (ANOVA) and Bonferroni’s test for pairwise comparisons. Unstimulated mutants were compared with each other (row ANOVA). Considering each cell line, the results obtained after TNF and IL-1β stimulation were compared with those from unstimulated cells (column ANOVA).

## Abbreviations

CRDs: Cysteine-rich domains
CRP: C-reactive protein
DD: Death domains
IL-1R: Interleukin 1 receptor
LPS: Lipopolysaccharide
PBMCs: Peripheral blood mononuclear cells
PBS: Phosphate buffered saline
SAA: Serum amyloid A
TNF: Tumor necrosis factor
TNF-R1: Tumor-necrosis factor receptor 1
TRAPS: Tumor necrosis factor receptor-associated periodic syndrome
WT: Wild-type
