# Supplementary material for: The novel S59P mutation in the TNFRSF1A gene identified in an adult onset TNF receptor associated periodic syndrome (TRAPS) constitutively activates NF-κB pathway
Source: Arthritis Res Ther. 2015 Apr 3;17(1):93. doi: 10.1186/s13075-015-0604-7 (PMC4416318; doi:10.1186/s13075-015-0604-7)
Supplement: Additional file 1: — List of primary antibodies used in TNF-R1 signalling study. For lysate microarray (RPPA) analysis, they were diluted as shown in the table. [file 13075_2015_604_MOESM1_ESM.pdf]

Table 2. List of primary antibodies used in TNF-R1 signalling study. All antibodies were rabbit antibodies and were purchased from Cell Signaling Technology.

TNF receptor 1 pathway

Antibody	dilution
A20/TNFAIP3	1:150
TRAF2	1:500
p-RIP2(Ser^{176})	1:500

NF-κB pathway

Antibody	dilution
IKKα	1:250
IKKβ	1:250
IKKγ	1:250
p-IKKα/β(Ser^{176}/Ser^{177})	1:250
Iκ-Bα	1:100
p-Iκ-Bα(Ser^{32})	1:500
NF-κB	1:250
p-NF-κB(Ser^{536})	1:100

PI3K/AKT pathway

Antibody	dilution
SHIP2	1:2,000
p-eNOS(Ser^{1177})	1:500
p-AKT(Thr^{308})	1:50
p-AKT(Ser^{473})	1:25
PI3K p100a	1:250
PI3K p85	1:250
p-PDK1(Ser^{241})	1:500
p-GSK3b(Ser^{9})	1:500
p-PTEN(Ser^{380})	1: 1,000

MAP kinase pathway

Antibody	dilution
p-p38 MAPK(Thr^{180}/Tyr^{182})	1:1,000
p-MKK3/MKK6(Ser^{189}/Ser^{207})	1:100
p-HSP27(Ser^{82})	1:50

c-Jun pathway

Antibody	dilution
p-SAPK/JNK(Thr^{183}/Tyr^{185})	1:1,000
MKK7	1:100
p-c-JUN(Ser^{63})	1:200

ERK pathway

Antibody	dilution
p-c-RAF(Ser^{259})	1:1,000
MEK1/2	1:250
p-MEK1/2(Ser^{217}/Ser^{221})	1:250
p-ERK1/2(Thr^{202}/Tyr^{204})	1:2,000

SRC/JAK/STAT pathway

Antibody	dilution
p-STAT3(Tyr^{705})	1:100
p-c-SRC(Tyr^{416})	1:10,000
SOCS3	1:500
p-JAK2(Tyr^{1007})	1:1,000

IL-1β pathway

Antibody	dilution
MyD88	1:50
p-IRAK1(Thr^{209})	1:3,000
TRAF6	1:500
p-TAK1(Ser^{412})	1:100

Inflammasome pathway

Antibody	dilution
p-STAT1(Tyr^{701})	1:250
p-STAT4(Tyr^{693})	1:100
ASC	1:150
Caspase 1	1:250
CLEAVED Caspase 1(Asp^{297})	1:50
NALP1	1:150

Apoptosis pathway

Antibody	dilution
BCL-XL	1:250
BCL-2	1:250
p-BCL-2 (Ser^{70})	1:100
p-BAD(Ser^{136})	1:500
