# Supplementary material for: The novel S59P mutation in the TNFRSF1A gene identified in an adult onset TNF receptor associated periodic syndrome (TRAPS) constitutively activates NF-κB pathway
Source: Arthritis Res Ther. 2015 Apr 3;17(1):93. doi: 10.1186/s13075-015-0604-7 (PMC4416318; doi:10.1186/s13075-015-0604-7)
Supplement: Additional file 2: — RPPA data obtained from unstimulated, TNF and IL-1β stimulated wild-type (WT) and mutant HEK-293 cells. For any target, data are reported as mean ± SE (two independent experiments) percentage changes in fluorescence intensity, with respect of WT-HEK293 unstimulated cells. The statistical analysis was made by one-way analysis of variance (ANOVA) and Bonferroni’s test for pairwise comparisons. Unstimulated mutants were compared with each other (row ANOVA). Considering each cell line, the results obtained after TNF and IL-1β stimulation were compared with those from unstimulated cells (column ANOVA). [file 13075_2015_604_MOESM2_ESM.pdf]

Table 3. RPPA data obtained from unstimulated, TNF and IL-1β stimulated WT and mutants HEK-293 cells. For any target, data are reported as mean ±SE (two independent experiments) percentage changes in fluorescence intensity with respect WT-HEK293 unstimulated cells. The statistical analysis was made by one-way analysis of variance (ANOVA) and Bonferroni's test for pairwise comparisons. Unstimulated mutants were compared with each other (row ANOVA). Considering each cell line, the results obtained after TNF and IL-1β stimulation were compared with those from unstimulated cells (colomn ANOVA).

**TNF receptor 1 pathway**						
**Target**	**Stimulation**	**WT Mean±SE**	**S59P Mean±SE**	**R92Q Mean±SE**	**T50M Mean±SE**	**Row ANOVA**
TRAF2	None	100±0	104.83±4.40	83.63±6.84	84.38±2.97	F=6.23, p = 0.055.
	TNF	92.05±1.30*	100.14±4.51	117.53±1.34*	96.83±0.60	
	IL-1β	87.48±0.59*	92.56±2.76	105.43±2.55	102.05±1.83*	
	**Column ANOVA**	F=59.34, p=0.004	F=2.43, p =0.236	F=16.09, p=0.025	F=19.67, p=0.019	
p RIP2 (Ser^{176})	None	100±0	150.67±28.05	75.74±7.09	139.39±21.40	F=3.73, p = 0.118
	TNF	85.50±9.60	123.87±22.44	80.87±5.00	122.30±6.89	
	IL-1β	81.16±1.99	122.24±2.06	81.82±3.83	135.79±10.83	
	**Column ANOVA**	F=3.04, p=0.189	F=0.59, p=0.607	F=0.36, p=0.726	F=0.39, p=0.707	
*: p<0.05 with respect to unstimulated cells						

**NF-κB pathway**						
**Target**	**Stimulation**	**WT Mean±SE**	**S59P Mean±SE**	**R92Q Mean±SE**	**T50M Mean±SE**	**Row ANOVA**
p IKKα/β (Ser^{176}/Ser^{177})	None	100±0	87.63±1.49	62.29±0.38§§	91.21±3.48	F=72.13, p=0.0006
	TNF	90.23±0.10*	93.64±3.56	77.68±0.09	71.28±2.03	
	IL-1β	104.88±1.04*	81.08±3.54	76.63±5.14	91.11±3.21	
	**Column ANOVA**	F=152.92, p=0.001*	F=4.30, p=0.131	F=8.34, p=0.06	F=14.89, p=0.03	
p Iκ-Bα (Ser^{32})	None	100±0	132.62±6.16§	77.14±0.75§§	115.85±0.69	F=57.24, p = 0.001
	TNF	88.53±1.07	154.11±2.50	81.59±7.67	74.83±1.10*	
	IL-1β	122.73±6.98	106.03±0.07*	79.43±1.16	120.61±2.52	
	**Column ANOVA**	F=18.18, p=0.021	F=39.39, p=0.007	F=0.24, p = 0.798	F=235, p<0.001	
*: p<0.05 with respect to unstimulated cells<br>§: p<0.05 with respect to WT-HEK293 cells<br>§§: p<0.05 with respect to WT-HEK293 cells and the other mutants						

**PI3K/AKT pathway**						
**Target**	**Stimulation**	**WT Mean±SE**	**S59P Mean±SE**	**R92Q Mean±SE**	**T50M Mean±SE**	**Row ANOVA**
SHIP2	None	100±0	106.74±10.60	72.70±3.31	93.24±2.44	F=6.70, p = 0.0487
	TNF	87.67±1.68	102.86±14.04	87.82±.507	99.93±0.45	
	IL-1β	108.08±17.73	95.94±1.04	75.86±3.75	111.59±5.47	
	**Column ANOVA**	F=1.00, p=0.465	F=0.29, p=0.767	F=7.54, p =0.067	F=7.16, p=0.072	
p eNOS (Ser^{1177})	None	100±0	100.77±5.23	67.57±0.26§	94.68±1.14	F=34.28, p=0.0026

	TNF	86.98±1.01	99.04±1.51	85.74±2.21*	86.66±2.29	
	IL-1β	77.97±3.47*	82.17±1.24	81.81±1.48*	92.84±3.45	
	**Column ANOVA**	F=28.18 p=0.011	F=10.16, p=0.046	F=38.57, p=0.007	F=2.87, p=0.201	
p AKT(Thr308)	None	100±0	116.36±1.01§§	71.95±1.23§§	131.94±0.29§§	F=1007.88, p < 0.0001
	TNF	87.56±1.51	122.32±9.25	84.15±2.64	83.24±5.71*	
	IL-1β	112.24±8.22	102.84±0.89	82.71±2.59	121.08±5.36	
	**Column ANOVA**	F=6.54, p=0.081	F=3.42, p=0.168	F=8.77, p=0.055	F=31.95, p=0.009	
p AKT(Ser473)	None	100±0	88.67±5.39	61.60±1.74§§	108.75±5.13	F=28.75, p =0.0036
	TNF	89.06±1.74	96.25±0.56	71.11±2.71	86.53±0.15*	
	IL-1β	85.10±5.08	74.25±0.76	65.89±2.61	106.05±1.21	
	**Column ANOVA**	F=6.19, p=0.0861	F=12.49 p=0.035	F=3.97, p=0.143	F=15.88, p=0.025	
PI3K p100a	None	100±0	74.87±0.71§	74.83±3.14§	81.26±2.91§	F=30.12, p = 0.0033
	TNF	86.70±1.65*	84.11±0.61	87.81±5.43	76.35±1.72	
	IL-1β	86.93±0.19*	62.77±2.27*	83.93±1.01	82.45±3.38	
	**Column ANOVA**	F=63.17, p=0.003	F=46.74, p=0.005	F=3.29, p=0.175	F=1.37, p=0.377	
p PDK1 (Ser241)	None	100±0	108.30±4.42	83.43±8.56	83.14±1.72	F=6.55, p = 0.051
	TNF	84.92±2.73*	101.30±1.42	99.09±2.79	89.16±2.29	
	IL-1β	100.69±1.02	83.32±2.12*	86.47±4.71	103.00±10.64	
	**Column ANOVA**	F=28.02, p=0.011	F=19.16, p=0.019	F=2.00, p=0.280	F=2.56, p=0.224	
p GSK3β (Ser9)	None	100±0	102.61±2.54	79.10±4.34§§	98.72±0.27	F=18.36, p =0.0084
	TNF	92.09±0.72*	110.59±2.66	87.25±3.57	81.72±0.23*	
	IL-1β	106.59±1.16*	81.57±1.19*	85.07±2.30	109.57±2.07*	
	**Column ANOVA**	F=84.99 p=0.0023	F=45.11 p=0.006	F=0.145, p=0.363	F=134.44 p=0.001	
p PTEN (Ser380)	None	100±0	136.44±2.48§§	88.10±1.68§§	101.29±0.97	F=173.38, p=0.0001.
	TNF	92.06±3.62	133.26±0.95	100.42±7.18	90.24±8.60	
	IL-1β	107.04±1.06	127.73±3.34	95.17±4.82	104.18±12.54	
	**Column ANOVA**	F=11.85, p=0.037	F=3.20, p=0.180	F=1.48, p=0.357	F=0.70, p=0.563	

*: p<0.05 with respect to unstimulated cells
§: p<0.05 with respect to WT-HEK293 cells
§§: p<0.05 with respect to WT-HEK293 cells and the other mutants

MAP kinase pathway

**Target**	**Stimulation**	**WT Mean±SE**	**S59P Mean±SE**	**R92Q Mean±SE**	**T50M Mean±SE**	**Row ANOVA**
p MKK3/MKK6 (Ser189/Ser207)	None	100±0	165.54±6.30§	90.54±3.53	136.25±17.37	F=13.44, p=0.015
	TNF	84.08±6.52	149.70±0.46	89.81±1.22	94.17±7.11	
	IL-1β	80.66±3.79	105.92±2.89*	79.32±3.52	104.25±7.35	
	**Column ANOVA**	F=5.63, p=0.096	F=59.22, p=0.003	F=4.48, p=0.125	F=3.57, p=0.161	
p	None	100±0	118.06±4.23	83.98±2.83§§	117.69±2.82	F=31.51,

HSP27(Ser[82])						p = 0.003
	TNF	70.94±6.42	106.17±1.17	93.79±4.09	74.12±4.89*	
	IL-1β	71.81±5.55	88.91±0.24*	68.17±3.13	98.96±0.19	
	**Column ANOVA**	F=11.37, p = 0.039	F=33.38, p=0.009	F=14.53, p=0.029	F=44.93, p=0.006	
*: p<0.05 with respect to unstimulated cells<br>§: p<0.05 with respect to WT-HEK293 cells<br>§§: p<0.05 with respect to WT-HEK293 cells and the other mutants						
**c-Jun pathway**						
**Target**	**Stimulation**	**WT Mean±SE**	**S59P Mean±SE**	**R92Q Mean±SE**	**T50M Mean±SE**	**Row ANOVA**
p SAPK/JNK (Thr[183]/Tyr[185])	None	100±0	138.31±4.39	80.56±2.92	162.37±13.71§	F=25.24, p = 0.0046
	TNF	136.94±7.15*	127.19±4.81	101.06±0.61	165.68±6.18	
	IL-1β	124.41±1.33	241.97±8.16*	120.32±8.17*	126.82±3.76	
	**Column ANOVA**	F=0.65, p =0.583	F=110.09, p =0.002	F=15.67, p =0.025	F=5.79, p=0.093	
MKK7	None	100±0	131.28±9.89	105.29±8.69	92.92±17.61	F=2.31, p = 0.218
	TNF	95.22±9.21	131.00±0.40	111.88±5.49	92.21±14.23	
	IL-1β	106.14±7.33	122.63±6.08	103.05±1.92	130.05±10.30	
	**Column ANOVA**	F=0.65, p =0.583	F=0.54, p =0.631	F=0.58, p =0.613	F=2.27, p=0.251	
*: p<0.05 with respect to unstimulated cells<br>§: p<0.05 with respect to WT-HEK293 cells						
**ERK pathway**						
**Target**	**Stimulation**	**WT Mean±SE**	**S59P Mean±SE**	**R92Q Mean±SE**	**T50M Mean±SE**	**Row ANOVA**
p c-RAF (Ser[259])	None	100±0	88.24±4.12	97.51±8.78	92.35±1.31	F=1.16, p=0.428
	TNF	97.54±4.74	126.72±4.10	103.72±6.02	98.39±5.89	
	IL-1β	110.67±9.80	79.58±13.87	93.70±1.87	113.16±0.90	
	**Column ANOVA**	F=1.23, p=0.406	F=8.35, p=0.059	F=0.66, p=0.580	F=9.22, p=0.053	
MEK1/2	None	100±0	110.09±20.87	75.36±1.29	114.29±10.16	F=2.25, p=0.224
	TNF	80.18±3.71*	81.55±3.65	82.57±4.38	96.61±7.16	
	IL-1β	81.43±1.73*	88.65±0.94	68.65±14.67	99.10±6.22	
	**Column ANOVA**	F=22.03, p=0.016	F=1.47, p=0.358	F=0.61, p=0.597	F=1.42, p=0.368	
ERK1/2	None	100±0	68.44±2.32§§	48.36±4.98§§	97.06±0.34	F=79.94, p = 0.0005
	TNF	141.22±7.86	118.27±2.62 *	111.31±6.32*	125.87±16.47	
	IL-1β	102.14±14.04	85.72±2.94	80.59±0.63*	112.51±0.19	
	**Column ANOVA**	F=6.24, p =0.085	F=92.06, p=0.002	F=45.56, p=0.006	F=2.30, p=0.248	
*: p<0.05 with respect to unstimulated cells<br>§§: p<0.05 with respect to WT-HEK293 cells and the other mutants						
**SRC/JAK/STAT pathway**						
**Target**	**Stimulation**	**WT Mean±SE**	**S59P Mean±SE**	**R92Q Mean±SE**	**T50M Mean±SE**	**Row ANOVA**
p STAT3 (Tyr[705])	None	100±0	121.57±6.56	74.70±6.75§§	124.94±2.64	F=22.55, p =0.006
	TNF	92.39±2.52	110.43±4.24	78.00±6.54	85.52±7.68*	

	IL-1β	98.44±11.65	111.00±13.07	78.63±5.33	105.79±0.81	
	**Column ANOVA**	F=0.34, p=0.735	F=0.51, p=0.645	F=0.11, p=0.895	F=17.52 p=0.022	
p c-SRC (Tyr^{416})	None	100±0	111.73±18.46	82.34±2.79	113.26±2.81	F=2.29, p = 0.220.
	TNF	73.95±7.90	123.82±1.92	70.67±4.15	105.30±9.98	
	IL-1β	107.31±18.92	91.07±12.63	73.61±5.72	129.64±10.56	
	**Column ANOVA**	F=2.19, p=0.258	F=1.63, p=0.331	F=1.92, p=0.291	F=2.11, p=0.268	
p JAK2 (Tyr^{1007})	None	100±0	139.12±3.46§§	94.69±8.20	124.91±2.91	F=20.05, p =0.0071
	TNF	96.14±3.63	152.89±1.88	97.02±4.58	104.34±1.34*	
	IL-1β	125.98±18.37	134.04±3.51	92.87±7.84	124.06±1.28	
	**Column ANOVA**	F=2.25, p=0.252	F=10.26, p=0.046	F=0.09, p=0.919	F=34.14 p=0.009	

*: p<0.05 with respect to unstimulated cells
§§: p<0.05 with respect to WT-HEK293 cells and the other mutants

IL-1β pathway

**Target**	**Stimulation**	**WT Mean±SE**	**S59P Mean±SE**	**R92Q Mean±SE**	**T50M Mean±SE**	**Row ANOVA**
MyD88	None	100±0	115.71±8.15	81.08±8.7	118.40±9.89	F=4.92, p = 0.078
	TNF	90.82±5.10	118.01±0.14	99.24±15.85	91.30±11.97	
	IL-1β	105.30±1.27	90.62±0.67	99.30±3.43	100.85±3.86	
	**Column ANOVA**	F=5.82, p = 0.092.	F=10.37 P = 0.045	F=0.98, p = 0.470.	F=2.21 P = 0.257.	
TRAF6	None	100±0	107.96±5.98	95.85±17.19	109.94±15.83	F=0.30, p < 0.823
	TNF	93.42±3.85	113.02±16.83	115.97±5.02	102.77±9.47	
	IL-1β	99.45±0.53	78.89±0.80	79.17±0.12	110.65±16.32	
	**Column ANOVA**	F=2.64, p=0.218	F=3.18, p=0.181	F=3.18, p=0.181	F=0.09, p=0.913	
p TAK1 (Ser^{412})	None	100±0	126.38±4.91§§	92.94±4.86	107.26±3.26	F=14.2, p<0.013
	TNF	87.02±2.59*	123.98±9.60	109.57±2.79	78.26±3.68	
	IL-1β	100.97±0.20	127.52±8.09	95.24±4.24	130.53±23.09	
	**Column ANOVA**	F=27.05, p=0.01 *	F=0.05, p=0.948	F=4.93, p=0.112	F=3.69, p=0.155	

*: p<0.05 with respect to unstimulated cells
§§: p<0.05 with respect to WT-HEK293 cells and the other mutants

Inflammasome pathway

**Target**	**Stimulation**	**WT Mean±SE**	**S59P Mean±SE**	**R92Q Mean±SE**	**T50M Mean±SE**	**Row ANOVA**
p STAT1 (Tyr^{701})	None	100±0	154.07±6.58	90.77±16.21	120.55±18.73	F=4.79, p = 0.082
	TNF	104.07±8.64	134.38±4.18	92.22±0.17	108.37±11.03	
	IL-1β	101.00±14.91	110.08±13.86	94.57±10.09	131.13±3.49	
	**Column ANOVA**	F=0.05, p=0.956	F=5.76, p=0.093	F=0.03, p=0.970	F=0.80, p=0.525	
p STAT4 (Tyr^{693})	None	100±0	116.26±5.29	63.24±1.21	109.90±15.47	F=8.36, p = 0.034
	TNF	81.09±3.41	93.54±12.93	61.41±2.24	82.37±2.76	
	IL-1β	76.51±16.10	130.31±9.75	70.73±3.10	89.46±11.80	

	**Column ANOVA**	F=1.72, p=0.318	F=3.56, p=0.161	F=4.56, p=0.123	F=1.59, p=0.338	
ASC	None	100±0	114.26±1.29	95.99±1.91	103.04±7.56	F=3.94, p = 0.109
	TNF	83.05±3.81	103.79±0.25*	87.58±4.69	92.98±1.31	
	IL-1β	88.31±6.49	80.96±1.14*	83.14±6.60	126.28±1.75	
	**Column ANOVA**	F=3.99, p=0.142	F=286.3, p<0.001	F1.84, p=0.301	F=14.15, p=0.029	
CLEAVED Caspase 1 (Asp^{297})	None	100±0	93.11±0.82	80.32±0.70	116.22±6.41§§	F=21.17, p = 0.0064
	TNF	100.63±0.77	104.78±9.17	86.83±0.45*	113.89±8.32	
	IL-1β	102.07±12.18	71.40±2.85	87.12±0.50*	124.48±5.13	
	**Column ANOVA**	F=0.02, p=0.97	F=9.27, p=0.052	F=46.74, p=0.005	F=0.68, p=0.570	
NALP1	None	100±0	80.35±2.49	83.77±1.49	107.07±7.11	F=11.12, p = 0.021
	TNF	79.08±0.45	81.87±10.87	94.48±9.96	92.26±7.58	
	IL-1β	93.05±9.61	71.74±0.33	84.68±2.21	94.95±3.32	
	**Column ANOVA**	F=3.68, p=0.156	F=0.72, p=0.555	F=1.0, p=0.466	F=1.57, p=0.341	
*: p<0.05 with respect to unstimulated cells<br>§§: p<0.05 with respect to WT-HEK293 cells and the other mutants						

**Apoptosis pathway**						
**Target**	**Stimulation**	**WT Mean±SE**	**S59P Mean±SE**	**R92Q Mean±SE**	**T50M Mean±SE**	**Row ANOVA**
BCL-XL	None	100±0	89.43±1.15	67.72±1.03	123.23±25.32	F=331, p = 0.138
	TNF	83.96±1.10	87.61±3.27	57.17±8.14	80.51±0.12	
	IL-1β	79.59±10.30	64.64±0.39*	66.45±2.71	101.02±6.86	
	**Column ANOVA**	F=3.23, p=0.178	F=46.98, p=0.005	F=1.33, p =0.385	F=1.99, p =0.282	
BCL-2	None	100±0	239.33±91.70	218.77±3.05	157.74±0.62	F=1.89, p = 0.009
	TNF	174.08±7.87*	343.89±19.40	200.20±12.80	168.42±21.32	
	IL-1β	195.70±11.39*	399.19±26.65	166.82±6.30	154.05±9.71	
	**Column ANOVA**	F=39.42, p=0.007	F=2.08, p=0.270	F=9.77, p=0.048	F=0.30, p =0.758	
p BAD(Ser^{136})	None	100±0	161.93±23.41	99.70±2.10	113.94±14.25	F=4.59, p = 0.087
	TNF	86.64±6.17	128.96±0.34	111.27±3.75	108.46±0.67	
	IL-1β	96.45±14.60	107.88±3.75	95.98±3.34	149.82±27.75	
	**Column ANOVA**	F=0.57, p=0.616	F=3.96, p=0.144	F=6.43, p=0.082	F=1.56, p=0.344	
*: p<0.05 with respect to unstimulated cells						
